# Integration of ATAC-Seq and RNA-Seq Reveals the Role of *FaTIP1* in Red Light-Induced Fruit Ripening in Strawberry

**DOI:** 10.3390/ijms26020511

**Published:** 2025-01-09

**Authors:** Xiaodong Chen, Weijian Cai, Jin Xia, Jing Wang, Huazhao Yuan, Qinglian Wang, Fuhua Pang, Mizhen Zhao, Yushan Qiao

**Affiliations:** Institute of Pomology, Jiangsu Academy of Agricultural Sciences/Jiangsu Key Laboratory for Horticultural Crop Genetic Improvement/Jiangsu Province Engineering Research Center of Modern Strawberry Industry/Zhongshan Biological Breeding Laboratory, 50 Zhonglin Road, Nanjing 210014, China; 20160055@jaas.ac.cn (X.C.);

**Keywords:** aquaporin, LED light, strawberry, chromatin accessibility

## Abstract

Light is an important environmental factor affecting the ripening and quality of strawberry fruit. Previous studies have shown that red light treatment can promote strawberry ripening. Gene expression is closely associated with chromatin openness, and changes in chromatin accessibility are crucial for the binding of transcription factors to downstream regulatory sequences. However, the changes in chromatin accessibility in response to different light treatments in octoploid strawberry plants are still unclear. In this study, the landscape of chromatin accessibility of octoploid strawberry under red (R) and yellow–green (YG) light conditions was analyzed by the assay for transposase-accessible chromatin with high-throughput sequencing (ATAC-seq). Through bioinformatics and Venn diagram analyses, a total of 1456 and 1854 group-specific genes (GSGs) were screened in the R and YG groups, respectively. By using RNA sequencing (RNA-seq), 440 differentially expressed genes (DEGs) were identified. Among these genes, 194 were upregulated under red light treatment. Through joint analysis of ATAC-seq and RNA-seq data, three red group-specific genes with increased expression were identified, namely, *FaTIP1*, *FaQKY* and *FaLBD1*. Through gene expression and transient transformation analyses of strawberry fruit, we further demonstrated that *FaTIP1* can respond to red light induction and promote the ripening process of strawberry fruit. Our results provide a reference for the study of chromatin accessibility in octoploid strawberry and reveal new factors involved in the fruit’s response to red light and the regulation of the ripening process of strawberry fruit.

## 1. Introduction

The strawberry (*Fragaria × ananassa* Duch.) is a perennial herb of the genus *Fragaria* in the Rosaceae family. Due to its unique flavor and high nutritional value, strawberry is a popular fruit worldwide. The strawberry has a short growth cycle, and is an important economic fruit crop that is grown in multiple countries worldwide [1,2]. The ripening time and size of the fruit directly affect the economic benefits of planting strawberry [1].

Strawberry is a nonclimacteric soft fruit [3]. In the early stages of strawberry fruit development, there are mainly two biological processes in strawberry fruit: an increase in cell number, and cell enlargement [4,5,6]. The process of cell expansion depends mainly upon the transport of water into vacuoles [3]. Auxin and cytokinin play important roles in the early stages of strawberry fruit development [1]. In the later stage of strawberry fruit development, the auxin and gibberellin content gradually decreases, which allows the fruit to enter the mature stage from the growth and development stage [4]. During the mature stage, the water content of strawberry fruit accounts for more than 90% of the fresh weight [3]. The ripening process of strawberry fruit is regulated mainly by abscisic acid [4,5].

Water transport in plants mainly involves the apoplastic pathway and symplastic pathway [7]. The plasma membrane provides a barrier between the external environment and the cell [8,9], and water is transported in plants through self-diffusion and passive transport [10,11]. Passive transport is the main pathway by which water enters and exits cells, and the water channels formed by water pore proteins, which are present on the plasma membrane, mediate the passive transport of water between different plasma membranes [12]. Aquaporins (AQPs, often called aquaporin water channels) are a family of small, integral membrane proteins that are found in all living organisms, from bacteria to humans [13]. They are involved in the transmembrane diffusion of water, as well as various small solutes [14,15]. Aquaporins belong to the large family of membrane intrinsic proteins (MIPs), and MIPs in plants can be divided into four categories: plasma membrane intrinsic proteins (PIPs), tonoplast intrinsic proteins (TIPs), NOD26-like intrinsic proteins (NIPs) homologous to soybean NOD26, and small basic intrinsic proteins (SIPs) [12,15,16].

Research has shown that AQPs are widely involved in many aspects of plant growth and development [15,17], and some studies have revealed the function of AQPs in fruit development [15]. In pear fruit, TIPs are the most abundant proteins [18], and the gene encoding the TIP protein in pear (*Py-γTIP*) is enriched and expressed at the middle and end of the cell division stage of fruit development [19]. In peach, the *Pr-gTIP1* gene is highly expressed in the early stages of fruit development and the final stage of fruit growth, and there is a certain correlation between the expression level of *Pr-gTIP1* and the fruit growth and development rate [20,21]. Nir Sade et al. [22] reported that the overexpression of *SlTIP2;2* in tomato significantly increased the fruit yield, harvest index and plant mass. Several studies have revealed the function of AQPs in strawberry fruit development. Gabriela Amodeo et al. reported that there is a certain correlation between the expression levels of *FaPIP1* and *FaPIP2* in strawberry fruit and the firmness of the fruit [23,24]. Francisco J. Molina-Hidalgoa et al. showed that *FaNIP1;1* was specifically expressed in the receptacle and correlated with the fruit ripening process [3]. Although many studies have revealed the relationship between AQPs and fruit development, the specific function of AQPs in fruit development is still unknown.

The development of strawberry fruit involves processes such as growth, expansion and coloration. Light is an important factor affecting the growth, development and fruit ripening in strawberry [4,5]. With the development of LED lighting technology, the application of plant LED lighting technology in production and scientific research is increasing [25]. Light quality is an important characteristic, and has a significant impact on the quality and ripening of strawberry. In a previous study, we found that blue LED light treatment promoted the synthesis of chlorogenic acid and lutein in strawberry [26,27]. Research has also revealed that red LED light treatment can promote the ripening of strawberry fruit by accelerating the synthesis of anthocyanins [28,29,30].

In this study, by using a combination of the assay for transposase-accessible chromatin with high-throughput sequencing (ATAC-seq) and RNA sequencing (RNA-seq), we screened out the water pore protein-encoding gene *FaTIP1*. The expression of *FaTIP1* was induced by red light. Moreover, through transient transformation of strawberry fruit, we demonstrated that *FaTIP1* plays an important role in promoting strawberry ripening.

## 2. Results

### 2.1. Red Light Treatment Promotes the Development of Strawberry Fruit

**Figure 1:**
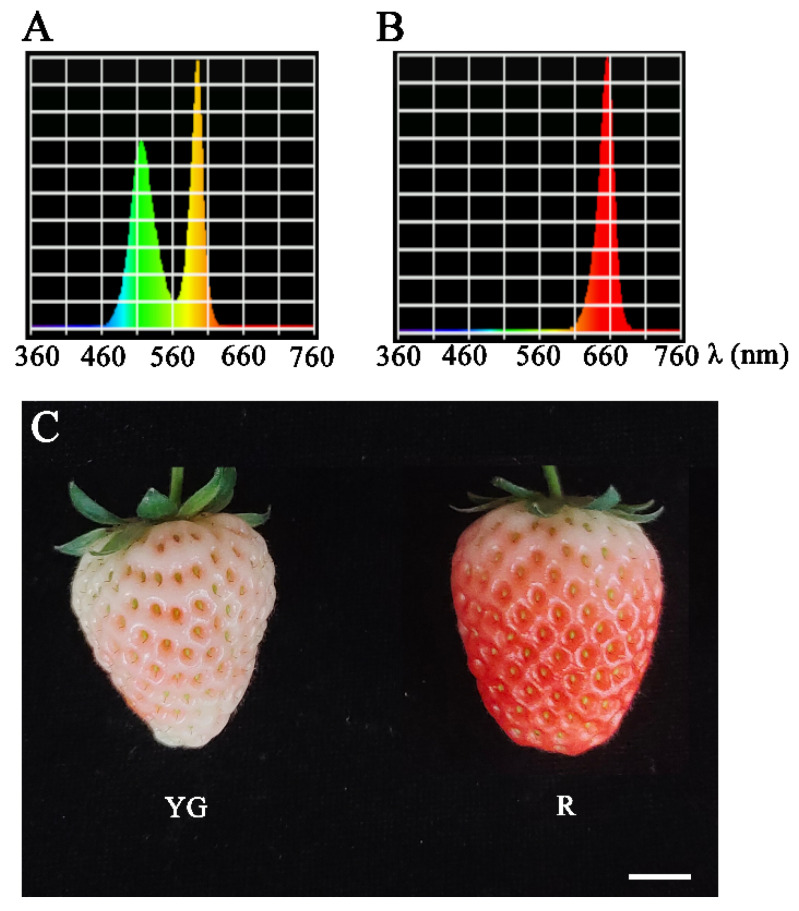
Ripening of strawberry fruits under different light treatments. (**A**,**B**) Electromagnetic spectrum of yellow–green light and red light, respectively. (**C**) Strawberry fruits after 15 days of light treatment. Scale bars, 1 cm. YG, yellow–green light; R, red light.

To study the effects of different wavelengths of light on strawberry development, LED luminescence technology was used to study the effects of yellow–green (YG) light and red (R) light treatments on strawberry growth and development (Figure 1A,B). As shown in Figure 1C, after 15 days of treatment, the strawberry fruit subjected to red light treatment had basically completed the fruit coloration process and entered the red fruit stage, while those subjected to yellow–green light treatment were still in the color transformation stage. These results indicate that red light treatment can promote the ripening of strawberry fruit.

### 2.2. Chromatin Accessibility Dynamics in Strawberry Fruit Under Different Light Treatments

To better visualize the changes in chromatin accessibility in response to different light treatments, ATAC-seq was applied to investigate the landscape of genomic chromatin accessibility changes in strawberry fruit under different light treatments. A total of 548,025,312 clean ATAC-seq reads were obtained for the different treatment groups (Table 1). High-quality (HQ) clean reads were obtained by removing low-quality reads, as well as reads containing adapters or more than 10% unknown nucleotides. These HQ clean reads were then aligned to the genome sequence. The results showed that the mappability was greater than 61%, and the percentage of unique mapped reads was 24% on average for all the samples. We identified 12,148 to 18,281 peaks in each ATAC-seq library (Table 1).

**Figure 2:**
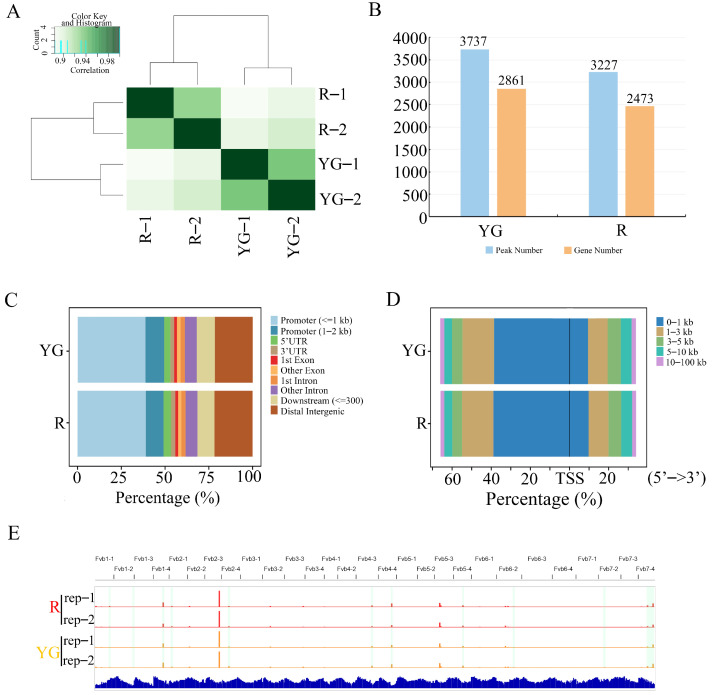
Chromatin accessibility landscape of strawberry fruit under different light treatment groups. (**A**) Correlation heatmap of four ATAC-seq samples. Darker green means higher correlation. (**B**) Numbers of peaks and genes in different light treatment groups. (**C**) Distribution proportion of peaks in gene functional elements of each sample. (**D**) Distribution proportion diagram of peaks relative to TSS for each sample. (**E**) Genome browser of the global ATAC-seq tracks of YG group and R group. The differential areas of chromatin accessibility between different samples are highlighted in light green shades. YG, yellow–green light; R, red light.

To further verify the reliability of the ATAC-seq data, we analyzed the correlations among the four samples. There was a strong correlation between the two biological replicates for each treatment (Figure 2A). We further filtered the peaks between duplicate samples within the group and retained the shared peaks with an overlap > 50% for subsequent analysis. Through the combined analysis of intragroup peaks, 3737 and 3227 peaks were ultimately identified in the YG and R groups, corresponding to 2861 and 2473 genes, respectively (Figure 2B and Table S3). These peaks were mainly distributed around transcription start sites (TSSs) (Figure 2D). Relative location analysis of the peaks revealed that they were mainly distributed in the promoter (0–2 kb) region (46.86% and 46.64% for YG and R, respectively) and distal intergenic region (21.76% and 21.88% for YG and R, respectively) (Figure 2C). The global ATAC-seq tracks of YG and R were visualized in the IGV browser. Notably, there were multiple regions with different degrees of chromatin accessibility in the two groups (Figure 2E).

### 2.3. Enrichment Analysis of Group-Specific Genes (GSGs) Under Different Light Treatments

**Figure 3:**
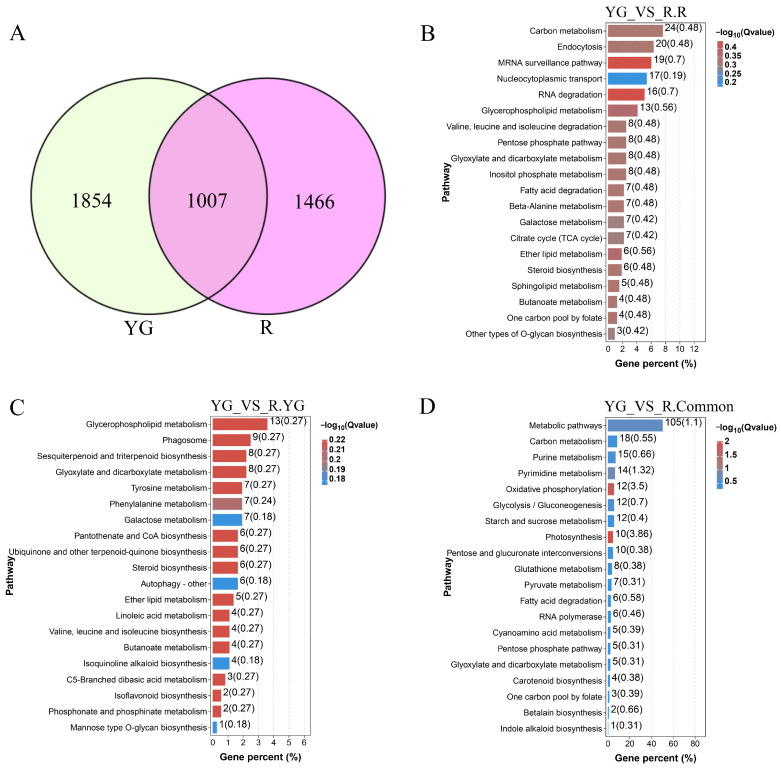
The KEGG pathway enrichment analysis of the genes identified by ATAC-seq in different groups. (**A**) Venn diagrams of genes corresponding to peaks identified by ATAC-seq between YG and R groups. (**B**,**C**) KEGG enrichment analysis of genes specifically identified in the R group and YG group, respectively. (**D**) KEGG enrichment analysis of common genes between YG and R. YG, yellow–green light; R, red light.

We further analyzed the genes specifically enriched in the R and YG groups by conducting Venn analysis on the genes corresponding to the peaks in the YG and R groups. A total of 1466 genes were unique to the R group, and 1854 genes were unique to the YG group (Figure 3A). Gene enrichment analysis revealed that the top five KEGG pathways enriched in the R group were involved in carbon metabolism, endocytosis, mRNA survival, RNA degradation and glycophoric metabolism (Figure 3B). This result indicates that the specific genes identified under red light treatment were involved mainly in carbon metabolism. The top five KEGG pathways enriched in the YG group were biosynthesis of amino acids, endocytosis, glycerophospholipid metabolism, glutathione metabolism and phagosome (Figure 3C), which suggested that the specific genes identified under yellow–green light treatment were involved mainly in amino acid metabolism. The results of KEGG enrichment analysis showed that the genes shared by the YG and R groups were involved mainly in carbon metabolism, purine and pyrimidine metabolism, oxidative phosphorylation, etc. (Figure 3D).

### 2.4. Motif Identification and Construction of Transcriptional Regulatory Networks for Strawberry Fruit Development Under Red Light Treatment

**Figure 4:**
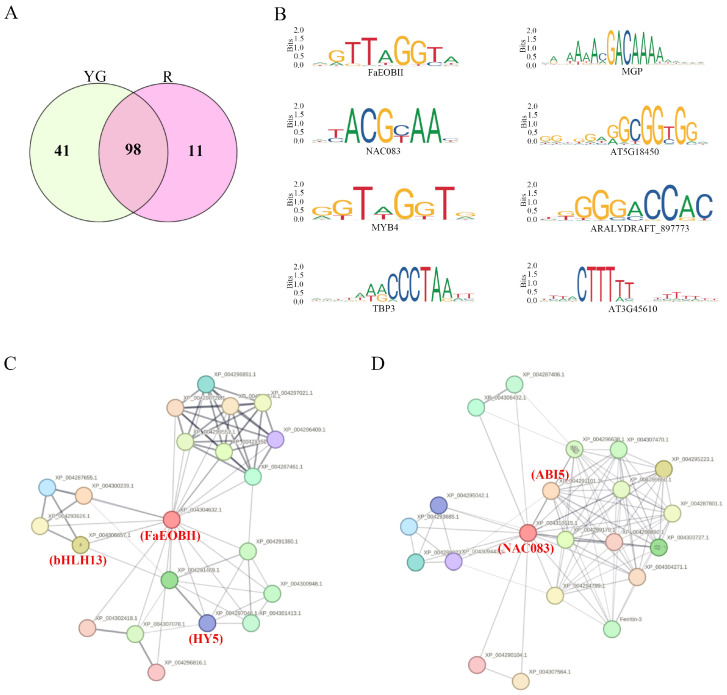
Analysis of transcription factor (TF) binding motifs in different groups. (**A**) Venn diagrams of transcription factor (TF) binding motifs between YG and R groups. (**B**) The sequence of transcription factor (TF) binding motifs that only appears in the R group. (**C**) STRING interaction diagram of the FaEOBII. (**D**) STRING interaction diagram of the NAC083. Line thickness indicates the strength of data support.

To identify specific transcription factors (TFs) that may play important roles in regulating fruit development under different light treatments, we identified overrepresented cis-regulatory motifs via MEME-ChIP. We identified 139 and 109 cis-regulatory motifs in the YG and R groups, respectively (Table S4). We performed Venn diagram analysis on the motifs identified in each group and screened for specific and shared motifs in each group. A total of 11 cis-regulatory motifs were found only in group R, and 41 motifs were specifically present in the YG group (Figure 4A). The 11 motifs in group R included those of MYB domain protein 4 (MYB4), the emission of benzenoid II (FaEOBII), MYB46, NAC domain-containing protein 83 (NAC083), TATA-box binding protein 3 (TBP3), MAGPIE (MGP)/INDETERMINATE DOMAIN 3 (IDD3), IDD4, IDD7, AT5G18450, ARALYDRAFT_897773 and AT3G45610 (Figure 4B and Figure S1). Among them, *FaEOBII* plays an important role in the development of strawberry fruit, participating in the synthesis of some aroma substances [31]. The *TBP* gene encodes a tropical DNA-binding protein [32]. *MGP* (*IDD3*), *IDD4* and *IDD7* belong to the C2H2 BIRD translation factor family [33]. Related studies have shown that the *MYB* and *NAC* transcription factors play important roles in the ripening process of strawberry fruit [34,35]. We conducted protein interaction network analysis on FaEOBII and NAC083, using the STRING database. The results showed that FaEOBII could interact with HY5, which is an important gene in the light-signaling pathway and positively regulates anthocyanin synthesis [36], and basic helix-loop-helix factor 13 (bHLH13) (Figure 4C and Table S5). NAC083 potentially interacts with ABI5 in strawberry (Figure 4D and Table S5). Studies have shown that ABI5 plays an important role in promoting the ripening of strawberry fruit [37]. In addition, IDD proteins coregulate many chromatin remodeling factors (Table S5). These results indicate that the FaEOBII, NAC083 and IDD proteins may play important roles in the response of strawberry fruit to red light-induced ripening.

### 2.5. Integrated ATAC-Seq and RNA-Seq Analysis

**Figure 5:**
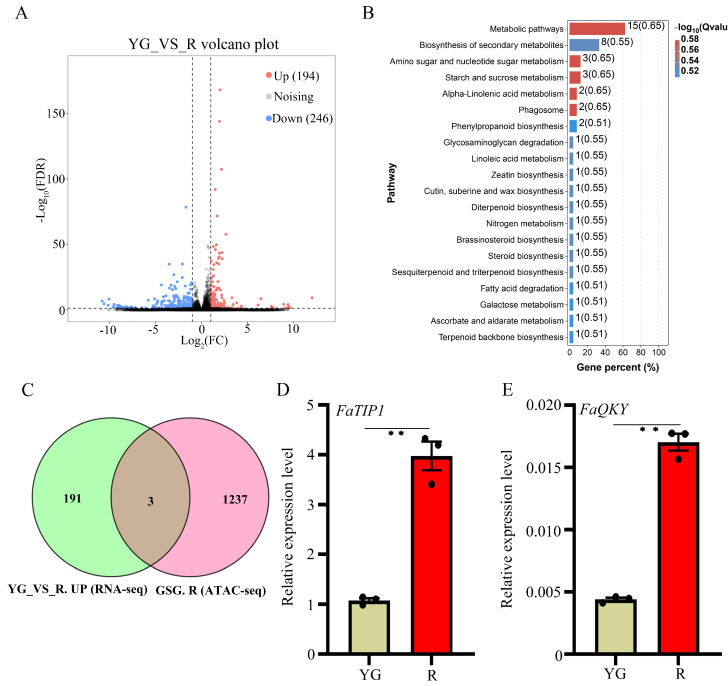
The association analysis of ATAC-seq and RNA-seq. (**A**) The gene number of up- and down-regulated genes identified by RNA-seq between YG and R groups. (**B**) KEGG enrichment analysis of up-regulated genes (RNA-seq) of the R group in the comparison of YG-VS-R. (**C**) The Venn diagram shows the overlaps between the up-regulated genes identified by RNA-seq and group-specific genes (GSG) identified by ATAC-seq in the R group. YG-VS-R.R-ATAC: the group-specific genes identified in the R group. YG-VS-R.UP-RNA-seq: the up-regulated genes of R in the comparison of YG and R. (**D**,**E**) The expression level of *FaTIP1* and *FaQKY* under different light treatments. YG and R represent yellow–green and red light, respectively. Data represent the mean ± SEM (*n* = 3). **, *p* < 0.01 in a two-sided Student’s *t*-test with the control. Scale bars, 1 cm.

To further investigate the mechanism by which red light treatment promotes strawberry fruit ripening, we used RNA-seq to analyze gene expression in strawberry fruit under different light quality treatments. By using fold change ≥ 2 and *q*-value ≤ 0.05 as thresholds, we identified 440 DEGs (194 upregulated and 246 downregulated) in YG_VS_R (Figure 5A and Table S6). KEGG enrichment analysis revealed that these upregulated genes were involved in starch and sucrose metabolism, galactose metabolism, amino sugar and nucleotide sugar metabolism, etc. (Figure 5B). Chromatin accessibility is closely related to gene expression. We used Venn diagram analysis to screen for genes specific to the R group in the ATAC-seq data and upregulated genes in the R group in the RNA-seq data. Finally, three genes that were highly expressed according to the transcriptome data and were specific to group R were screened. These three genes were *TIP1* (Fvb6-2-augustus-gene-167.32), *QUIRKY* (*QKY*, Fvb5-3-processed-gene-63.6) [38] and LATERAL ORGAN BOUNDARIES DOMAIN protein 1 (*LBD1*, maker-Fvb7-3-augustus-gene-82.54) [39] (Figure 5C). In plants, *TIP1* encodes a tonoplast intrinsic protein that functions as a water channel [40], QKY is a protein with multiple C2 domains and transmembrane regions [38], and *LBD1* belongs to the *LBD* gene family, members of which act as key regulators of plant organ development [39]. We also used qRT–PCR to verify the expression of these three genes. The results showed that the expression levels of *FaTIP1* and *FaQKY* under red light treatment were 3.7 and 3.9 times higher, respectively, than those in the YG group (Figure 5D,E), which was consistent with the RNA-seq data. However, there was no significant difference in the expression level of *FaLBD1* between the R and YG groups, according to the qRT–PCR results (Figure S2).

### 2.6. Functional Analysis of FaTIP1 and FaQKY

**Figure 6:**
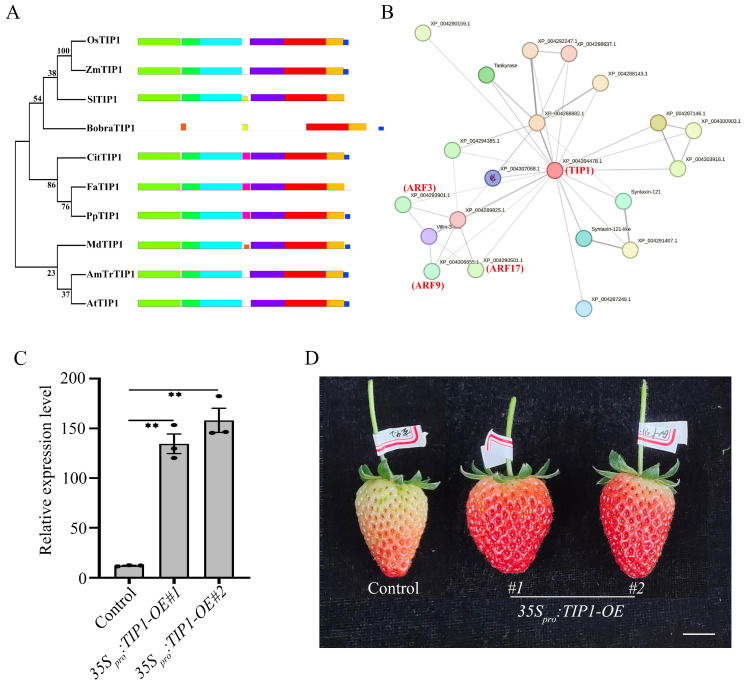
The functional analysis of TIP1 in strawberry. (**A**) Genetic evolution analysis of ten TIP1 proteins retrieved from *Botryococcus braunii* (*Bobra*), *Amborella trichopoda* (*AmTr*), *Zea mays* (*Zm*), *Oryza sativa* (*Os*), *Arabidopsis thaliana* (*At*), *Prunus persica* (*Pp*), *Solanum lycopersicum* (*Sl*), *Malus domestica* (*Md*), *Citrus clementina* (*Cit*) and *Fragaria ananassa* (*Fa*). The protein motifs are denoted by rectangles of different colors. The sequences of these motifs are shown in Figure S4. (**B**) STRING interaction diagram of the FaTIP1. (**C**) The expression level of *FaTIP1* in transiently transformed fruits. Data represent the mean ± SEM (*n* = 3). **, *p* < 0.01 in a two-sided Student’s *t*-test with the control. (**D**) Fruits after 11 days of infiltration. Scale bars, 1 cm.

We constructed evolutionary trees for the TIP1 and QKY proteins of 10 crop species, namely, *Botryococcus braunii* (*Bobra*), *Amborella trichopoda* (*AmTr*), *Zea mays* (*Zm*), *Oryza sativa* (*Os*), *Arabidopsis thaliana* (*At*), *Prunus persica* (*Pp), Solanum lycopersicum* (*Sl*), *Malus domestica* (*Md*), *Citrus clementina* (*Cit*) and *Fragaria ananassa* (*Fa*). The results showed that FaTIP1 was more closely related to peach and citrus, while FaQKY was more closely related to peach and apple (Figure 6A and Figure S3). MEME analysis of the conserved motifs of these proteins revealed that all the TIP1 proteins, except that from *B. braunii*, had a similar conserved functional domain, while the protein structure of QKY was conserved mainly in higher plants (Figure 6A and Figure S3).

To verify the functions of *FaTIP1* and *FaQKY* in the development of strawberry fruit, we used a transient transformation method to overexpress *FaTIP1* and *FaQKY* in strawberry fruit. The results showed that overexpression of *FaTIP1* in strawberry could promote fruit development, while overexpression of *FaQKY* did not accelerate fruit ripening (Figure 6C,D and Figure S3). The STRING database was used to analyze the protein interaction network of TIP1. TIP1 exhibited a potential relation with auxin-signaling pathway molecules, including AUXIN RESPONSE FACTOR 3 (ARF3), ARF9 and ARF17 (Figure 6B). ARF proteins are key factors in the auxin-signaling pathway and are involved in the processes of cell growth and expansion [41]. The correlation between TIP1 and ARF proteins also suggested that TIP1 plays an important role in the process of cell growth and expansion.

## 3. Discussion

Plant factory systems are extensively used worldwide, and many crops, such as lettuce, tomato and strawberry, have been successfully planted in plant factories [42,43,44]. Light is an important environmental factor for regulating plant growth [45]. Different plants have different requirements for artificial light sources, so the selection of artificial light sources for use in plant factories is a very important consideration [27]. In this study, ATAC-seq, combined with RNA-seq, revealed that red light treatment can induce the expression of *FaTIP1*. Further studies showed that overexpression of *FaTIP1* could promote the ripening process of strawberry fruit. Taken together, our results suggested that red light might promote the ripening process of strawberry fruits under red light treatment by inducing the expression of the aquaporin-encoding gene *FaTIP1* in strawberry. These findings reveal a new function of light in regulating the development of strawberry fruit and provide a theoretical basis for the development and scientific use of light for strawberry cultivation in the future.

ATAC-seq is an important technology that has been developed in recent years for detecting chromatin accessibility, and it has been widely used in humans, plants, animals and yeasts [46,47,48]. In this study, 3737 and 3227 peaks were screened in the YG and R groups, respectively, which suggested that the change in chromatin accessibility was more significant in the YG group. However, the distribution of chromatin accessibility peaks in the genome was similar between the two groups, with most of the peaks enriched in the promoter and distal intergenic regions (Figure 2C,D).

Integrated analysis of ATAC-seq and RNA-seq data can reveal the relationship between chromatin accessibility and gene expression and provide useful insights based on high-throughput data analysis [48]. In this study, we identified 2861 and 2473 group-specific genes in the YG and R groups, respectively (Figure 2B; Table S3). However, only 440 DEGs (194 upregulated and 246 downregulated) were identified through RNA-seq (Figure 5A; Table S6). The number of DEGs identified by RNA-seq was significantly lower than the number of group-specific genes identified by ATAC-seq. This difference may have been caused by the use of octoploid strawberry as the experimental material in this study.

Through a joint analysis of ATAC-seq and RNA-seq data, we identified three genes with high expression specifically in the R group. Through gene expression and functional validation analyses, we further determined that *FaTIP1* plays an important role in the red light-induced ripening of strawberry fruit. TIP1 is a water channel protein located in the vacuolar membrane [40]. Vacuoles occupy 90% of the volume of mature cells, and fruit enlargement depends mainly on the increase in the volume of vacuoles in fruit cells [49,50]. The main component of vacuoles is water, and the amount of water determines the volume of fruit cells [3,21]. Hence, water channel proteins on the vacuole membrane are very important for cell volume expansion. The enlargement of strawberry fruit occurs mainly before the white fruit stage, after which the fruit enter the coloration stage [4]. Therefore, we speculated that *FaTIP1* may promote the transport of water to the fruit and thus promote the expansion and ripening of strawberry fruit.

Light and water are two important environmental factors for fruit growth and development [51]. The results showed that red light could induce the expression of *FaTIP1*. However, how light induces the expression of aquaporin-encoding genes is still unclear. Previous studies have shown that the expression of *AtTIP1;1* and *AtTIP5;1* is induced by gibberellic acid (GA) [52]. Red light can induce the biosynthesis of GA [53]. In addition, through motif enrichment analysis, we found that MGP (IDD3), IDD4, and IDD7 were specifically enriched in the red light treatment group. In peach, PpIDDs can act as cofactors of PpDELLA1, which then promotes the synthesis of GA by activating the expression of *PpGA20ox1* (a GA biosynthesis-related gene) [54]. Based on these findings, we speculate that red light may promote the expression of *FaTIP1* in strawberry plants through the GA and IDD signaling pathways, but the specific mechanism involved needs further study.

## 4. Materials and Methods

### 4.1. Plant Materials and Light Treatment

The material used in this project was the octoploid strawberry cultivar ‘Ningyu’. The strawberry planting and cultivation methods used were as described previously [27]. When the strawberry fruit reached the large green fruit stage, the seedlings were moved to n climate chamber with an artificial light source for different light treatments. The photoperiod included 10 h of illumination and 14 h of darkness, with a light intensity of 150 μmol m^−2^ s^−1^. The red light had a wavelength of 660 nm, and yellow–green light was emitted as a combination of yellow light with a wavelength of 590 nm and green light with a wavelength of 520 nm in a 1:1 ratio.

### 4.2. ATAC-Seq

ATAC-seq libraries were constructed according to the methods described by Buenrostro [55]. In brief, nuclear suspensions were incubated in a transposition mixture that included a transposase. Adapter sequences were added to the ends of the DNA fragments. The transposition reaction was conducted at 37 °C for 30 min. Immediately following transposition, the products were purified using a QIAGEN MiniElute Kit and sequenced on the Illumina HiSeqTM 4000 platform by Gene Denovo Biotechnology Co. (Guangzhou, China). Bowtie2 [56] (version 2.2.8) was used to align the clean reads from each sample against the reference genome, and the reads aligned to the mitochondria or chloroplasts were filtered. MACS [57] (version 2.1.2) was used for peak calling. The dynamic Poisson distribution was used to calculate the *p* value of the specific region based on the unique mapped reads. The region was defined as a peak when the *q* value was <0.05. ChIPseeker [58] (version v1.16.1) was used to confirm peak-related genes and the distribution of peaks in different genomic regions. The MEME Suite (http://meme-suite.org/, accessed on 15 September 2023) was used to detect the motifs [59]. We used MEME-ChIP [60] to scan motifs with high reliability through peak regions and used MEME-AME [61] to confirm the existence of any specific known motifs. The calculated *p* value was subjected to FDR correction, taking an FDR ≤ 0.05 as the threshold. Pathways meeting this condition were defined as significantly enriched pathways among the peak-related genes. For the KEGG enrichment analysis of group-specific genes, the calculated *p* value was subjected to FDR correction, taking an FDR ≤ 0.05 as the threshold. Pathways meeting this condition were defined as significantly enriched pathways among the peak-related genes.

### 4.3. RNA-Seq

The RNA-seq method used was described previously [27]. Briefly, total RNA was extracted from strawberry fruit treated with yellow–green and red light, and RNA-seq was performed using the Illumina HiSeq 2500 platform (Gene Denovo Biotechnology, Guangzhou, China). The reads were mapped to the *Fragaria* × *ananassa Camarosa* Genome Assembly v1.0.a1 [62] using HISAT2.2.4 [63]. For each transcription region, a fragment per kilobase of transcript per million mapped reads (FPKM) value was calculated to quantify its expression level and variations using StringTie software (v1.3.1) [64]. Differential RNA expression analysis was performed by DESeq2 R package (1.20.0) [65]. Genes/transcripts for which the FDR was ≤0.05 and the absolute fold change was ≥2 were considered differentially expressed genes (DEGs)/differentially expressed transcripts. Three biological replicates were used in the data analyses.

### 4.4. Quantitative Reverse-Transcription PCR (qRT–PCR) Analysis

qRT–PCR was carried out as previously described [27]. The qRT–PCR results are presented as relative transcript levels, normalized against that of *FaACTIN*. The primers used for qRT–PCR are listed in Table S1.

### 4.5. Bioinformatics Analysis

The strawberry FaTIP1 and FaQKY protein sequences were used as query sequences to perform BLASTP searches against the *Botryococcus braunii v2.1* [66], *Amborella trichopoda v1.0* [67], *Solanum lycopersicum ITAG5.0* [68], *Malus domestica v1.1* [69], *Prunus persica* v2.1 [70], *Arabidopsis thaliana TAIR10* [71], *Citrus clementina v1.0* [72], *Oryza sativa v7.0* [73] and *Zea mays PHB47 v1.2* [74] databases, using Phytozome 13 [75]. The sequence with the highest score was selected. Phylogenetic tree and protein domain analyses were performed, as previously described [27]. The STRING online database (version 12.0) [76] was used to predict potential protein–protein interaction networks. *Fragaria vesca* was selected as the reference organism for comparison. No more than 20 interactors were predicted.

### 4.6. Gene Cloning and Agrobacterium Infiltration

The *FaTIP1* and *FaQKY* coding sequences were inserted into the pMON530 vector to generate *35S_pro_:FaTIP1* and *35S_pro_:FaQKY*. Then, the constructs were introduced into Agrobacterium strain GV3101. Agrobacterium infiltration was performed as previously described [27].

## Figures and Tables

**Table 1:** Summary of the ATAC-seq data.

Sample_ID	Clean_Reads_Num	HQ_Clean_Reads_Num (%)	Mapped_Reads	Unique_Mapped_Reads	PeakNumber	FRiP
YG-1	156291818	139162400 (89.04%)	86056715-61.84%	32999849-23.71%	12,559	0.7951
YG-2	149168524	134179794 (89.95%)	82020476-61.13%	32359101-24.12%	15,631	0.8223
R-1	119962168	109943072 (91.65%)	67542833-61.43%	27378229-24.90%	12,148	0.8377
R-2	122602802	110064872 (89.77%)	68820702-62.53%	26716520-24.27%	18,281	0.8155

FRiP, Fraction of reads in peaks.

## Data Availability

The sequence data of TIP1, QKY and LBD1 can be accessed at the GDR (https://www.rosaceae.org/, accessed on 15 September 2023) under the following accession numbers: *TIP1* (Fvb6-2-augustus-gene-167.32), *QKY* (Fvb5-3-processed-gene-63.6) and *LBD1* (maker-Fvb7-3-augustus-gene-82.54). The sequences used for genetic evolution analysis of the TIP1 and QKY proteins can be accessed at the Phytozome database (https://phytozome-next.jgi.doe.gov, accessed on 15 September 2023). The accession numbers are listed in Table S2. The ATAC-seq and RNA-seq data have been deposited in the Gene Expression Omnibus (http://www.ncbi.nlm.nih.gov/geo, accessed on 24 July 2023) under the accession number PRJNA997764.

## Supplementary Materials

The following supporting information can be downloaded at: https://www.mdpi.com/article/10.3390/ijms26020511/s1.

## References

[1] Hernández-Martínez N.R., Blanchard C., Wells D., Salazar-Gutiérrez M.R. (2023). Current state and future perspectives of commercial strawberry production: A review. Sci. Hortic..

[2] Lei J.J., Jiang S., Ma R.Y., Xue L., Zhao J., Dai H.P. (2021). Current status of strawberry industry in China. Acta Hortic..

[3] Molina-Hidalgo F.J., Medina-Puche L., Gelis S., Ramos J., Sabir F., Soveral G., Prista C., Iglesias-Fernández R., Caballero J.L., Muñoz-Blanco J. (2015). Functional characterization of FaNIP1;1 gene, a ripening-related and receptacle-specific aquaporin in strawberry fruit. Plant Sci..

[4] Li B.-J., Grierson D., Shi Y., Chen K.-S. (2022). Roles of abscisic acid in regulating ripening and quality of strawberry, a model non-climacteric fruit. Hortic. Res..

[5] Moya-León M.A., Mattus-Araya E., Herrera R. (2019). Molecular Events Occurring During Softening of Strawberry Fruit. Front. Plant Sci..

[6] Kang C., Darwish O., Geretz A., Shahan R., Alkharouf N., Liu Z. (2013). Genome-scale transcriptomic insights into early-stage fruit development in woodland strawberry Fragaria vesca. Plant Cell.

[7] Benitez-Alfonso Y. (2014). Symplastic intercellular transport from a developmental perspective. J. Exp. Bot..

[8] Heilmann M., Heilmann I. (2022). Regulators regulated: Different layers of control for plasma membrane phosphoinositides in plants. Curr. Opin. Plant Biol..

[9] Gronnier J., Gerbeau-Pissot P., Germain V., Mongrand S., Simon-Plas F. (2018). Divide and Rule: Plant Plasma Membrane Organization. Trends Plant Sci..

[10] Scharwies J.D., Dinneny J.R. (2019). Water transport, perception, and response in plants. J. Plant Res..

[11] Prieto I., Armas C., Pugnaire F.I. (2012). Water release through plant roots: New insights into its consequences at the plant and ecosystem level. New Phytol..

[12] Kaldenhoff R., Ribas-Carbo M., Sans J.F., Lovisolo C., Heckwolf M., Uehlein N. (2008). Aquaporins and plant water balance. Plant Cell Environ..

[13] Azad A.K., Raihan T., Ahmed J., Hakim A., Emon T.H., Chowdhury P.A. (2021). Human Aquaporins: Functional Diversity and Potential Roles in Infectious and Non-infectious Diseases. Front. Genet..

[14] Takata K., Matsuzaki T., Tajika Y. (2004). Aquaporins: Water channel proteins of the cell membrane. Prog. Histochem. Cytochem..

[15] Wang Y., Zhao Z., Liu F., Sun L., Hao F. (2020). Versatile Roles of Aquaporins in Plant Growth and Development. Int. J. Mol. Sci..

[16] Kapilan R., Vaziri M., Zwiazek J.J. (2018). Regulation of aquaporins in plants under stress. Biol. Res..

[17] Singh R.K., Deshmukh R., Muthamilarasan M., Rani R., Prasad M. (2020). Versatile roles of aquaporin in physiological processes and stress tolerance in plants. Plant Physiol. Biochem..

[18] Shiratake K., Kanayama Y., Maeshima M., Yamaki S. (1998). Changes in tonoplast protein and density with the development of pear fruit. Physiol. Plant..

[19] Shiratake K., Kobae Y., Suzuki Y., Nakaune M., Tanase K., Yamaki S. (2001). Molecular Cloning of a cDNA Encoding Tonoplast Water Channel of Pear Fruit and Its Expression during Development. Engei Gakkai Zasshi.

[20] Sugaya S., Gemma H., Iwahori S. (2001). Isolation and Expression Analysis of a Gene Encoding a Vacuolar-type Water Channel Protein in Peach Fruit. Engei Gakkai Zasshi.

[21] Shiratake K., Martinoia E. (2007). Transporters in fruit vacuoles. Plant Biotechnol..

[22] Sade N., Vinocur B.J., Diber A., Shatil A., Ronen G., Nissan H., Wallach R., Karchi H., Moshelion M. (2009). Improving plant stress tolerance and yield production: Is the tonoplast aquaporin SlTIP2;2 a key to isohydric to anisohydric conversion?. New Phytol..

[23] Alleva K., Marquez M., Villarreal N., Mut P., Bustamante C., Bellati J., Martínez G., Civello M., Amodeo G. (2010). Cloning, functional characterization, and co-expression studies of a novel aquaporin (FaPIP2;1) of strawberry fruit. J. Exp. Bot..

[24] Mut P., Bustamante C., Martínez G., Alleva K., Sutka M., Civello M., Amodeo G. (2008). A fruit-specific plasma membrane aquaporin subtype PIP1;1 is regulated during strawberry (*Fragaria* × *ananassa*) fruit ripening. Physiol. Plant..

[25] Kusuma P., Pattison P.M., Bugbee B. (2020). From physics to fixtures to food: Current and potential LED efficacy. Hortic. Res..

[26] Chen X., Cai W., Xia J., Yu H., Wang Q., Pang F., Zhao M. (2020). Metabolomic and Transcriptomic Analyses Reveal that Blue Light Promotes Chlorogenic Acid Synthesis in Strawberry. J. Agric. Food Chem..

[27] Chen X.-d., Cai W.-j., Xia J., Yuan H.-z., Wang Q.-l., Pang F.-h., Zhao M.-z. (2023). Metabolomic and transcriptomic analysis reveals the molecular mechanism by which blue light promotes lutein synthesis in strawberry. J. Integr. Agric..

[28] Zhang Y., Jiang L., Li Y., Chen Q., Ye Y., Zhang Y., Luo Y., Sun B., Wang X., Tang H. (2018). Effect of Red and Blue Light on Anthocyanin Accumulation and Differential Gene Expression in Strawberry (*Fragaria* × *ananassa*). Molecules.

[29] Zhang Y., Hu W., Peng X., Sun B., Wang X., Tang H. (2018). Characterization of anthocyanin and proanthocyanidin biosynthesis in two strawberry genotypes during fruit development in response to different light qualities. J. Photochem. Photobiol. B.

[30] Sánchez-Gómez C., Posé D., Martín-Pizarro C. (2022). Insights into transcription factors controlling strawberry fruit development and ripening. Front. Plant Sci..

[31] Medina-Puche L., Molina-Hidalgo F.J., Boersma M., Schuurink R.C., López-Vidriero I., Solano R., Franco-Zorrilla J.-M., Caballero J.L., Blanco-Portales R., Muñoz-Blanco J. (2015). An R2R3-MYB Transcription Factor Regulates Eugenol Production in Ripe Strawberry Fruit Receptacles. Plant Physiol..

[32] Mishal R., Luna-Arias J.P. (2022). Role of the TATA-box binding protein (TBP) and associated family members in transcription regulation. Gene.

[33] Feng X., Yu Q., Zeng J., He X., Ma W., Ge L., Liu W. (2023). Comprehensive Analysis of the INDETERMINATE DOMAIN (IDD) Gene Family and Their Response to Abiotic Stress in Zea mays. Int. J. Mol. Sci..

[34] Liu J., Wang J., Wang M., Zhao J., Zheng Y., Zhang T., Xue L., Lei J. (2021). Genome-Wide Analysis of the R2R3-MYB Gene Family in Fragaria × ananassa and Its Function Identification During Anthocyanins Biosynthesis in Pink-Flowered Strawberry. Front. Plant Sci..

[35] Martín-Pizarro C., Vallarino J.G., Osorio S., Meco V., Urrutia M., Pillet J., Casañal A., Merchante C., Amaya I., Willmitzer L. (2021). The NAC transcription factor FaRIF controls fruit ripening in strawberry. Plant Cell.

[36] Fu Z., Shang H., Jiang H., Gao J., Dong X., Wang H., Li Y., Wang L., Zhang J., Shu Q. (2020). Systematic Identification of the Light-quality Responding Anthocyanin Synthesis-related Transcripts in Petunia Petals. Hortic. Plant J..

[37] Li D., Mou W., Luo Z., Li L., Limwachiranon J., Mao L., Ying T. (2016). Developmental and stress regulation on expression of a novel miRNA, Fan-miR73, and its target ABI5 in strawberry. Sci. Rep..

[38] Song J.H., Kwak S.-H., Nam K.H., Schiefelbein J., Lee M.M. (2019). QUIRKY regulates root epidermal cell patterning through stabilizing SCRAMBLED to control CAPRICE movement in Arabidopsis. Nat. Commun..

[39] Grimplet J., Pimentel D., Agudelo-Romero P., Martinez-Zapater J.M., Fortes A.M. (2017). The LATERAL ORGAN BOUNDARIES Domain gene family in grapevine: Genome-wide characterization and expression analyses during developmental processes and stress responses. Sci. Rep..

[40] Rodrigues M.I., Takeda A.A.S., Bravo J.P., Maia I.G. (2016). The Eucalyptus Tonoplast Intrinsic Protein (TIP) Gene Subfamily: Genomic Organization, Structural Features, and Expression Profiles. Front. Plant Sci..

[41] Li Y., Han S., Qi Y. (2023). Advances in structure and function of auxin response factor in plants. J. Integr. Plant Biol..

[42] Nakayama M., Nakazawa Y. (2023). Effects of environmental control and LED supplemental lighting on strawberry growth and yield in a subtropical climate. Sci. Hortic..

[43] Jaeger S.R. (2024). Vertical farming (plant factory with artificial lighting) and its produce: Consumer insights. Curr. Opin. Food Sci..

[44] Zhang L., Yang X., Li T., Gan R., Wang Z., Peng J., Hu J., Guo J., Zhang Y., Li Q. (2022). Plant factory technology lights up urban horticulture in the post-coronavirus world. Hortic. Res..

[45] Xiao L., Shibuya T., Kato K., Nishiyama M., Kanayama Y. (2022). Effects of light quality on plant development and fruit metabolism and their regulation by plant growth regulators in tomato. Sci. Hortic..

[46] Sahu S.K., Basu A., Tiwari V.K. (2021). Measuring Chromatin Accessibility: ATAC-Seq. Methods Mol. Biol..

[47] Grandi F.C., Modi H., Kampman L., Corces M.R. (2022). Chromatin accessibility profiling by ATAC-seq. Nat. Protoc..

[48] Sun Z., Li J., Lv L., Gou Y., Wang B., Hao T. (2022). Integration of ATAC-seq and RNA-seq identifies active G-protein coupled receptors functioning in molting process in muscle of Eriocheir sinensis. Front. Mar. Sci..

[49] Hou X., Li H., Zhang W., Yao Z., Wang Y., Du T. (2021). Water transport in fleshy fruits: Research advances, methodologies, and future directions. Physiol. Plant..

[50] Tan X., Li K., Wang Z., Zhu K., Tan X., Cao J. (2019). A Review of Plant Vacuoles: Formation, Located Proteins, and Functions. Plants.

[51] Ali M.M., Yousef A.F., Li B., Chen F. (2021). Effect of Environmental Factors on Growth and Development of Fruits. Trop. Plant Biol..

[52] Pang Y., Li J., Qi B., Tian M., Sun L., Wang X., Hao F. (2018). Aquaporin AtTIP5;1 as an essential target of gibberellins promotes hypocotyl cell elongation in Arabidopsis thaliana under excess boron stress. Funct. Plant Biol..

[53] García-Martinez J.L., Gil J. (2001). Light Regulation of Gibberellin Biosynthesis and Mode of Action. J. Plant Growth Regul..

[54] Jiang Y., Chen J., Zheng X., Tan B., Ye X., Wang W., Zhang L., Li J., Li Z., Cheng J. (2022). Multiple indeterminate domain (IDD)-DELLA1 complexes participate in gibberellin feedback regulation in peach. Plant Mol. Biol..

[55] Buenrostro J.D., Giresi P.G., Zaba L.C., Chang H.Y., Greenleaf W.J. (2013). Transposition of native chromatin for multimodal regulatory analysis and personal epigenomics. Nat. Methods.

[56] Langmead B., Salzberg S.L. (2012). Fast gapped-read alignment with Bowtie 2. Nat. Methods.

[57] Zhang Y., Liu T., Meyer C.A., Eeckhoute J., Johnson D.S., Bernstein B.E., Nusbaum C., Myers R.M., Brown M., Li W. (2008). Model-based analysis of ChIP-Seq (MACS). Genome Biol..

[58] Yu G., Wang L.-G., He Q.-Y. (2015). ChIPseeker: An R/Bioconductor package for ChIP peak annotation, comparison and visualization. Bioinformatics.

[59] Bailey T.L., Johnson J., Grant C.E., Noble W.S. (2015). The MEME Suite. Nucleic Acids Res..

[60] Machanick P., Bailey T.L. (2011). MEME-ChIP: Motif analysis of large DNA datasets. Bioinformatics.

[61] McLeay R.C., Bailey T.L. (2010). Motif Enrichment Analysis: A unified framework and an evaluation on ChIP data. BMC Bioinform..

[62] Edger P.P., Poorten T.J., VanBuren R., Hardigan M.A., Colle M., McKain M.R., Smith R.D., Teresi S.J., Nelson A.D.L., Wai C.M. (2019). Origin and evolution of the octoploid strawberry genome. Nat. Genet..

[63] Kim D., Langmead B., Salzberg S.L. (2015). HISAT: A fast spliced aligner with low memory requirements. Nat. Methods.

[64] Pertea M., Pertea G.M., Antonescu C.M., Chang T.-C., Mendell J.T., Salzberg S.L. (2015). StringTie enables improved reconstruction of a transcriptome from RNA-seq reads. Nat. Biotechnol..

[65] Love M.I., Huber W., Anders S. (2014). Moderated estimation of fold change and dispersion for RNA-seq data with DESeq2. Genome Biol..

[66] Browne D.R., Jenkins J., Schmutz J., Shu S., Barry K., Grimwood J., Chiniquy J., Sharma A., Niehaus T.D., Weiss T.L. (2017). Draft Nuclear Genome Sequence of the Liquid Hydrocarbon-Accumulating Green Microalga Botryococcus braunii Race B (Showa). Genome Announc..

[67] Amborella Genome Project (2013). The Amborella genome and the evolution of flowering plants. Science.

[68] Zhou Y., Zhang Z., Bao Z., Li H., Lyu Y., Zan Y., Wu Y., Cheng L., Fang Y., Wu K. (2022). Graph pangenome captures missing heritability and empowers tomato breeding. Nature.

[69] Daccord N., Celton J.-M., Linsmith G., Becker C., Choisne N., Schijlen E., van de Geest H., Bianco L., Micheletti D., Velasco R. (2017). High-quality de novo assembly of the apple genome and methylome dynamics of early fruit development. Nat. Genet..

[70] Verde I., Jenkins J., Dondini L., Micali S., Pagliarani G., Vendramin E., Paris R., Aramini V., Gazza L., Rossini L. (2017). The Peach v2.0 release: High-resolution linkage mapping and deep resequencing improve chromosome-scale assembly and contiguity. BMC Genom..

[71] Lamesch P., Berardini T.Z., Li D., Swarbreck D., Wilks C., Sasidharan R., Muller R., Dreher K., Alexander D.L., Garcia-Hernandez M. (2012). The Arabidopsis Information Resource (TAIR): Improved gene annotation and new tools. Nucleic Acids Res..

[72] Wu G.A., Terol J., Ibanez V., López-García A., Pérez-Román E., Borredá C., Domingo C., Tadeo F.R., Carbonell-Caballero J., Alonso R. (2018). Genomics of the origin and evolution of Citrus. Nature.

[73] Ouyang S., Zhu W., Hamilton J., Lin H., Campbell M., Childs K., Thibaud-Nissen F., Malek R.L., Lee Y., Zheng L. (2007). The TIGR Rice Genome Annotation Resource: Improvements and new features. Nucleic Acids Res..

[74] Bornowski N., Michel K.J., Hamilton J.P., Ou S., Seetharam A.S., Jenkins J., Grimwood J., Plott C., Shu S., Talag J. (2021). Genomic variation within the maize stiff-stalk heterotic germplasm pool. Plant Genome.

[75] Goodstein D.M., Shu S., Howson R., Neupane R., Hayes R.D., Fazo J., Mitros T., Dirks W., Hellsten U., Putnam N. (2012). Phytozome: A comparative platform for green plant genomics. Nucleic Acids Res..

[76] Szklarczyk D., Kirsch R., Koutrouli M., Nastou K., Mehryary F., Hachilif R., Gable A.L., Fang T., Doncheva N.T., Pyysalo S. (2023). The STRING database in 2023: Protein-protein association networks and functional enrichment analyses for any sequenced genome of interest. Nucleic Acids Res..

