# Supplementary material for: Integration of ATAC-Seq and RNA-Seq Reveals the Role of FaTIP1 in Red Light-Induced Fruit Ripening in Strawberry
Source: Int J Mol Sci. 2025 Jan 9;26(2):511. doi: 10.3390/ijms26020511 (PMC11765184; doi:10.3390/ijms26020511)
Supplement: Supplementary file 1 [file ijms-26-00511-s001.zip › Supplementary materials.pdf]

Supplementary Materials

Table S1. List of primers used in this study.
Table S2. The accession numbers of TIP1 and QKY proteins.
Table S3. Peaks identified in YG and R groups.
Table S4. Motifs identified in YG and R groups.
Table S5. The protein interaction network of FaEOBII, NAC083, TIP1 and QKY in the STRING database.
Table S6. The DEGs between YG and R.
Figureure S1. The sequence of IDD4, IDD7 and MYB46.
Figure S2. The expression level of *FaLBD1* under different light treatments.
Figure S3. The functional analysis of QKY in strawberry.
Figure S4. The sequences of conserved motifs of TIP1 proteins identified by the MEME tool.
Figure S5. The sequences of conserved motifs of QKY proteins identified by the MEME tool.

Table S1. List of primers used in this study.

Gene Name	Primers	Primer Sequence (5' to 3')
FaTIP1-QRT	167.32-QRT-F1	CGGGCTGGTGTACACCGTCT
	167.32-QRT-R1	TTCTCCCAGCTCCAGCTCAC
FaQKY-QRT	63.6-QRT-F1	GCAGCAGCTACCTCAAGATG
	63.6-QRT-R1	AGGCGGAGATCTTGACGACG
FaLBD1-QRT	82.54-QRT-F1	CGAGACCCGGTTTACGGCTG
	82.54-QRT-R1	TCGAGTATGGTTGCTGCTGC
FaACTIN	FaACTIN-F1	TGGGTTTGCTGGAGATGAT
	FaACTIN-R1	CAGTAGGAGAACTGGGTGC

Table S2. The accession numbers of TIP1 and QKY proteins.

Name	Accession number
BobraTIP1	Bobra.0305s0005
AmTrTIP1	evm_27.TU.AmTr_v1.0_scaffold00102.27
SlTIP1	Solyc10G002669
MdTIP1	MD01G1121900
PpTIP1	Prupe.7G125900
AtTIP1	AT2G36830
CitTIP1	Ciclev10012553m.g
OsTIP1	LOC_Os03g05290
ZmTIP1	ZmPHB47.01G033800
BobraQKY	Bobra.0142s0045.1
AmTrQKY	evm_27.model.AmTr_v1.0_scaffold00102.27
SlQKY	Solyc01T001729.1
MdQKY	MD14G1004500
PpQKY	Prupe.7G007300.1
AtQKY	AT5G17980.1
CitQKY	Ciclev10018633m
OsQKY	LOC_Os07g07070.1
ZmQKY	ZmPHB47.07G023101.1

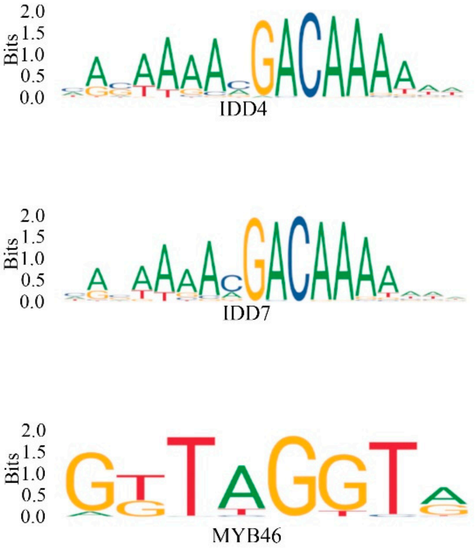

Figure S1. The sequence of IDD4, IDD7 and MYB46.

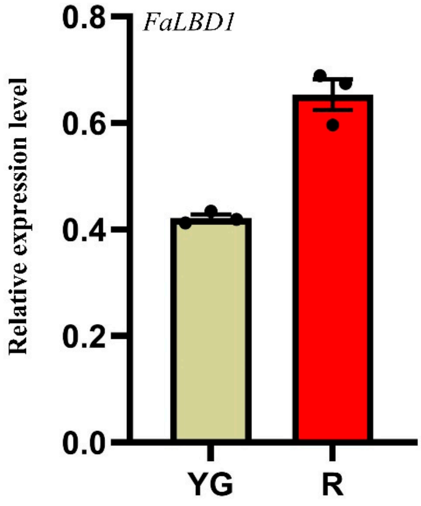

Figure S2. The expression level of *FaLBD1* under different light treatments. YG and R represent yellow-green and red light, respectively. Data represent the mean±SEM (n=3).

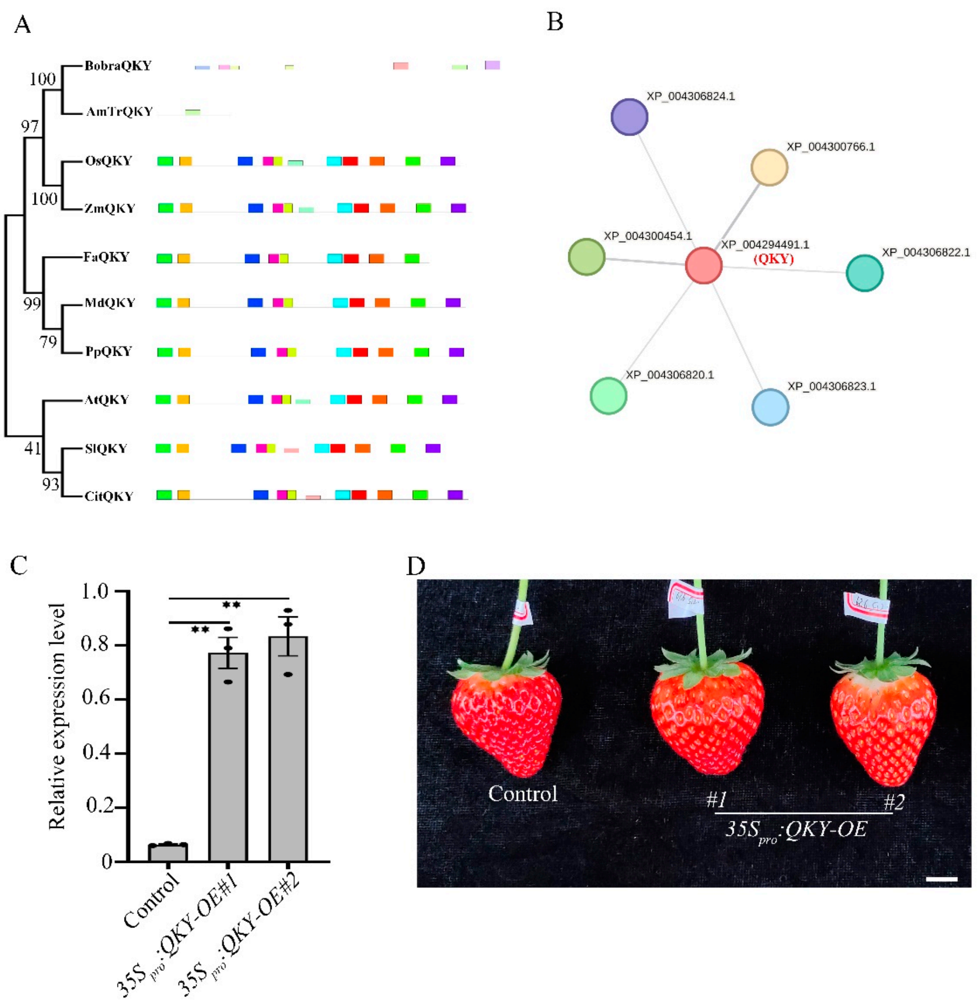

Figure S3. The functional analysis of QKY in strawberry. (A) Genetic evolution analysis of ten QKY proteins retrieved from *Botryococcus braunii* (*Bobra*), *Amborella trichopoda* (*AmTr*), *Zea mays* (*Zm*), *Oryza sativa* (*Os*), *Arabidopsis thaliana* (*At*), *Prunus persica* (*Pp*), *Solanum lycopersicum* (*Sl*), *Malus domestica* (*Md*), *Citrus clementina* (*Cit*) and *Fragaria ananassa* (*Fa*). The protein motifs are denoted by rectangles of different colors. The sequences of these motifs are shown in Figure. S5. (B) STRING interaction diagram of the FaQKY. (C) the expression level of *FaQKY* in transiently transformed fruits. Data represent the mean±SEM (n=3). ** , $P<0.01$ in a two-sided Student's t-test with the control. (D) Fruits after two weeks of infiltration. Scale bars, 1 cm.

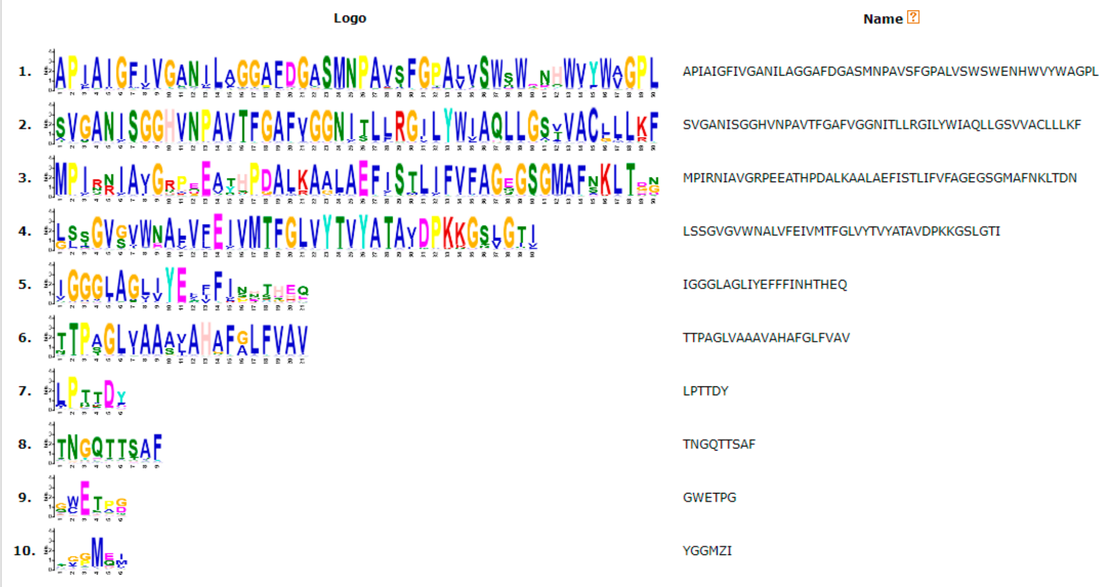

Figure S4. The sequences of conserved motifs of TIP1 proteins identified by the MEME tool.

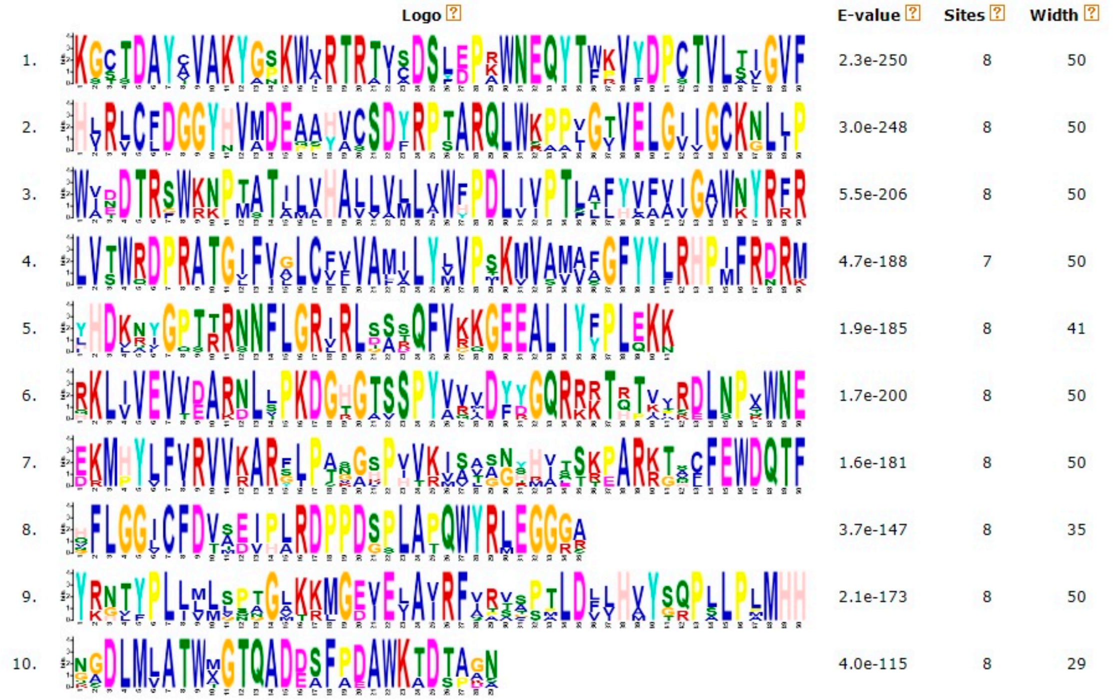

Figure S5. The sequences of conserved motifs of QKY proteins identified by the MEME tool.
